# Supplementary material for: Is e-cigarette use in non-smoking young adults associated with later smoking? A systematic review and meta-analysis
Source: Tob Control. 2020 Mar 10;30(1):8–15. doi: 10.1136/tobaccocontrol-2019-055433 (PMC7803902; doi:10.1136/tobaccocontrol-2019-055433)
Supplement: Supplementary data [file tobaccocontrol-2019-055433supp001.pdf]

Supplementary Material

Study Search

PubMed example:

(Tobacco Use[mesh] OR Tobacco[mesh] OR Tobacco use disorder[mesh] OR Tobacco Products[mesh] OR Cigar*[tiab] OR Tobacco[tiab] OR Smok*[tiab]) AND (Electronic Cigarettes[mesh] OR (Nebulizers and Vaporizers[mesh] AND (Tobacco[mesh] OR Tobacco[tiab] OR Nicotine[mesh] OR Nicotine[tiab])) OR Electronic Cigarette*[tiab] OR E-Cig*[tiab] OR Electronic Nicotine Delivery System*[tiab] OR Vape[tiab] OR Vaping[tiab] OR Alternative Nicotine Delivery System*[tiab])

Data Collection Process

For each paper, we extracted administrative details, study details and participant characteristics. Specifically, these included: author names; year of publication; country of the study; study design; study name (if applicable); sex of included participants, percentage of males included in the total sample and in the case and control groups; number of cases, controls and the size of the cohort; year(s) of data collection; age of the total sample, cases and controls; follow up length (if applicable); comparison group; exposure; outcome; covariates; definition of e-cigarette use and smoking; and type of assessment of e-cigarette use and smoking. We also extracted exposure and control details, outcome details, and results and conclusions. Specifically, these included: stratification information; direction of effect; effect estimate reported; number of individuals included in specific analyses; number of individuals exposed and unexposed in the analysis and number of subsequent smokers for each group; effect size, confidence intervals, standard errors and p-values for both unadjusted and adjusted analyses; and the conclusion regarding support for the gateway hypothesis.

Risk of Bias Assessment

Thresholds were applied to convert the Newcastle Ottowa Scale (NOS) for study quality to Agency for Health Research and Quality standards (whereby a good quality rating indicates low risk of bias and a poor rating indicates high risk of bias). Good quality ratings were determined by 3 or 4 stars in the selection domain and 1 or 2 stars in the comparability domain and 2 or 3 stars in the outcome/exposure domain. Fair quality was determined by 2 stars in the selection domain and 1 or 2 stars in the comparability domain and 2 or 3 stars in the outcome/exposure domain. Poor quality was determined by 0 or 1 star in the selection domain or 0 stars in the comparability domain or 0 or 1 stars in outcome/exposure domain.

Causality Criteria

Four Bradford-Hill criteria were selected to assess the evidence provided by the studies for a causal association: strength of association, specificity, temporality and dose responsivity. The specific thresholds and assessment techniques used are detailed below.

Strength of association. Strong associations were defined as having an adjusted odds ratio of two or more.

Specificity. Studies were considered specific if they adjusted for more than basic demographics (i.e. sex, age, socioeconomic position).

Temporality. The temporality criterion was met if studies were assessed longitudinally (i.e., e-cigarette use was measured at time point one with a measure of smoking prior to measuring later smoking at time point 2) – retrospective measures did not meet this criterion.

Dose Responsivity. Studies which measured and took into account frequency of e-cigarette use, length of time the product was used for, or how much nicotine was in the e-liquid used, were considered to meet the dose responsivity criterion.

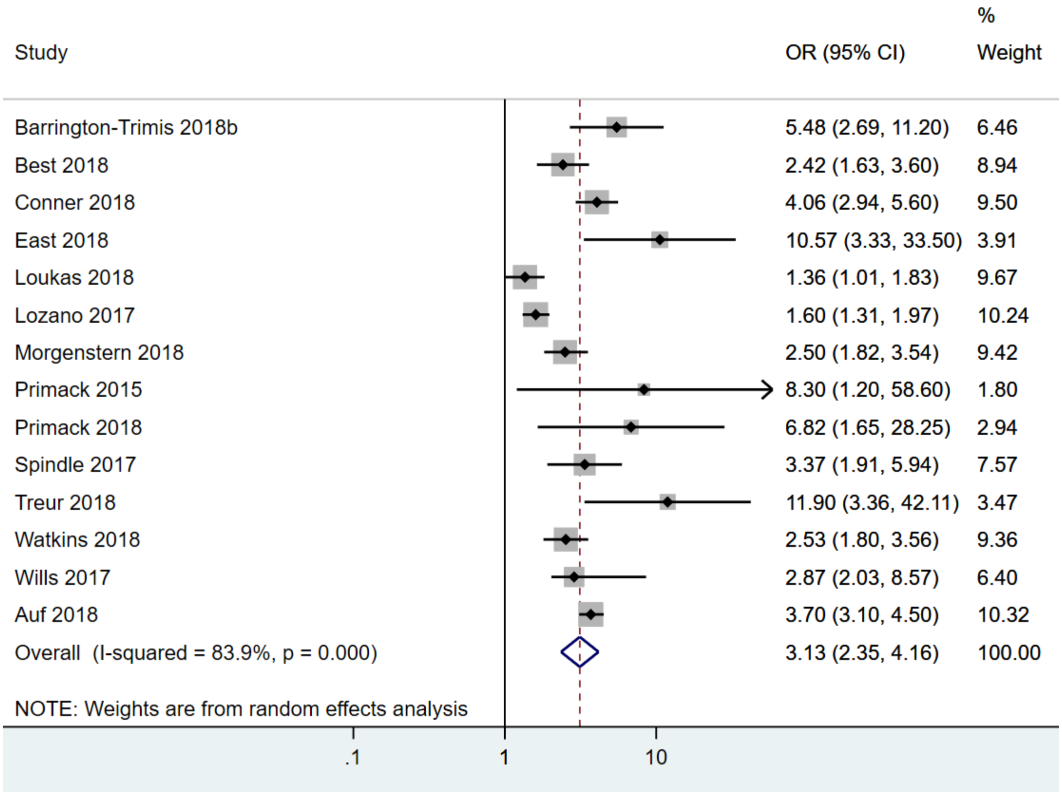

Figure S1. Forest plot for the adjusted association between ever e-cigarette use and later ever smoking.

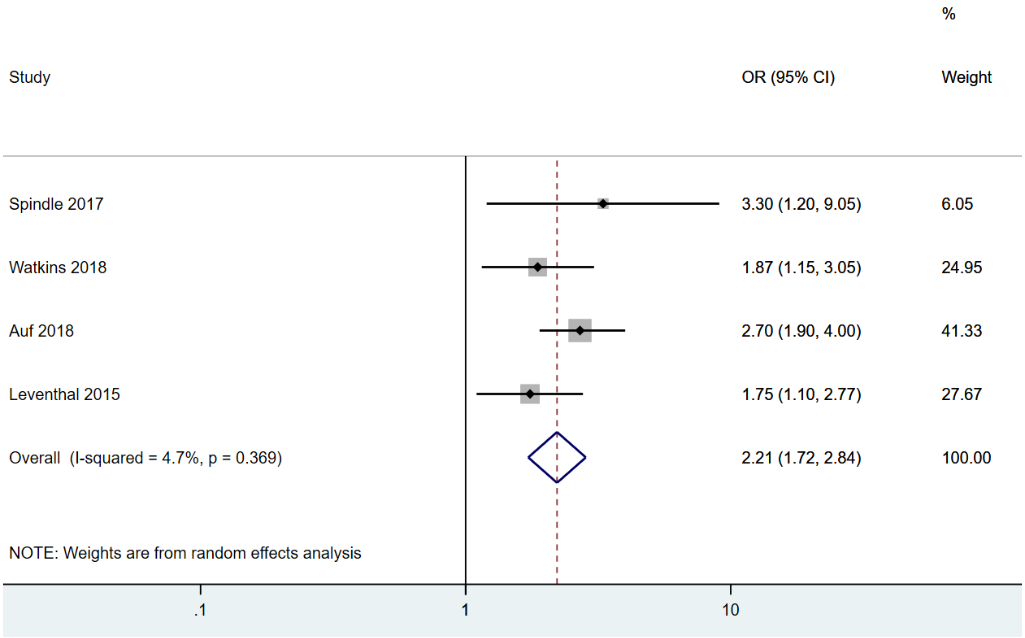

Figure S2. Forest plot for the adjusted association between ever e-cigarette use and later current smoking.

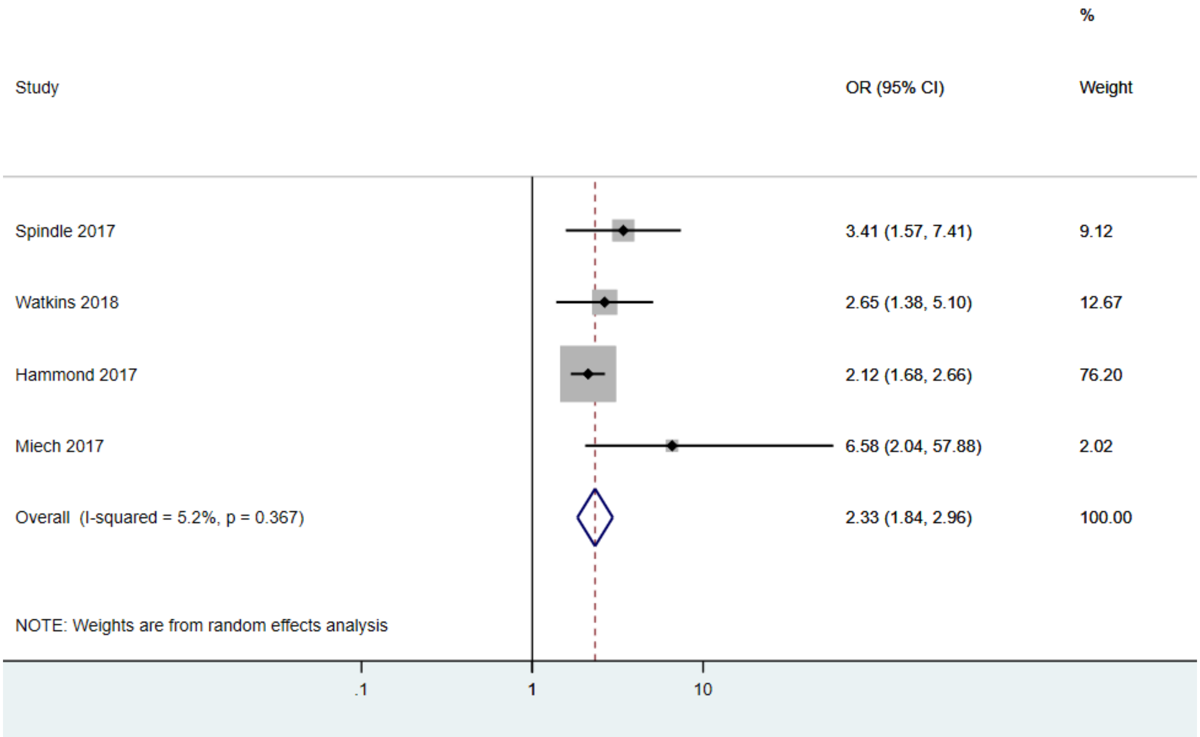

Figure S3. Forest plot for the adjusted association between past 30-day e-cigarette use and later ever smoking.

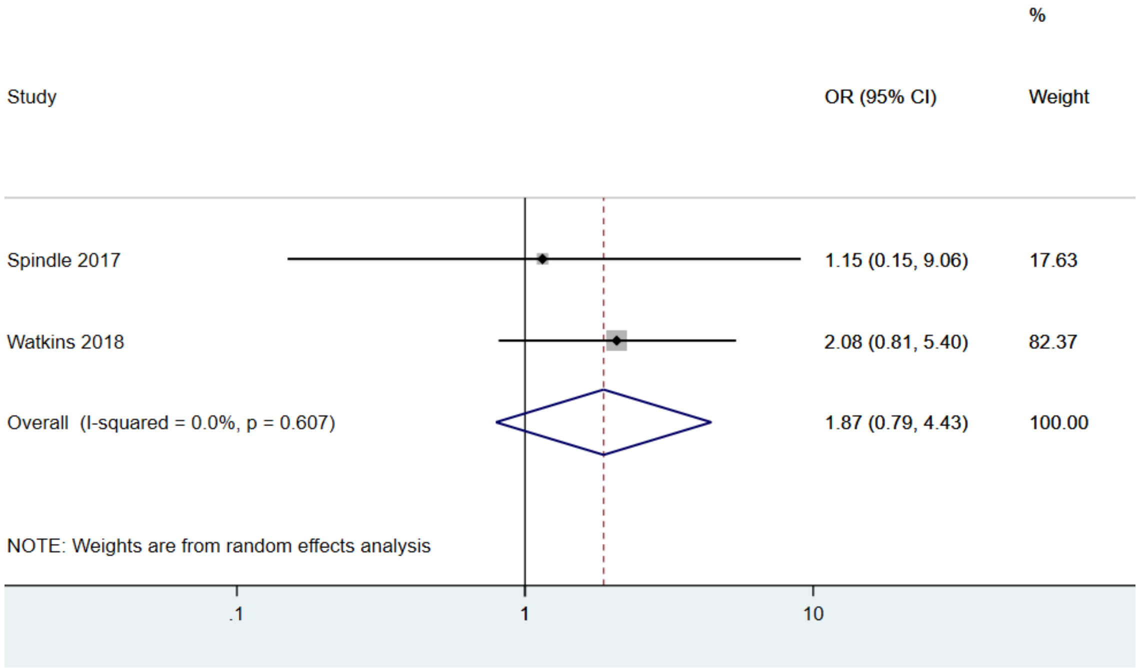

Figure S4. Forest plot for the adjusted association between current e-cigarette use and later current smoking.

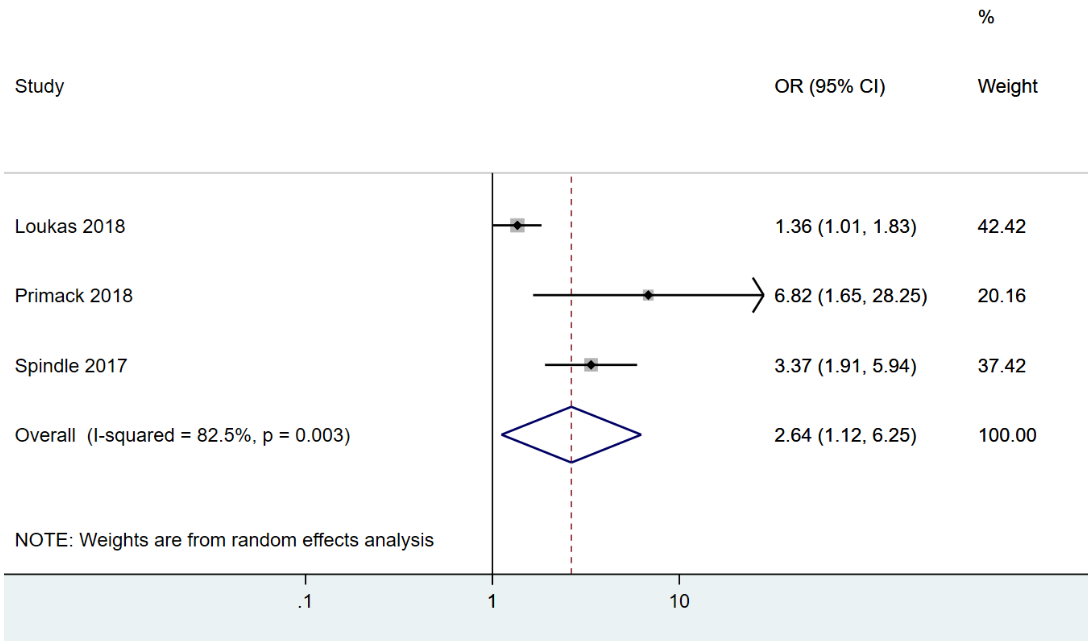

Figure S5. Forest plot for the adjusted association between e-cigarette use and later smoking among studies excluding under 18-year olds.

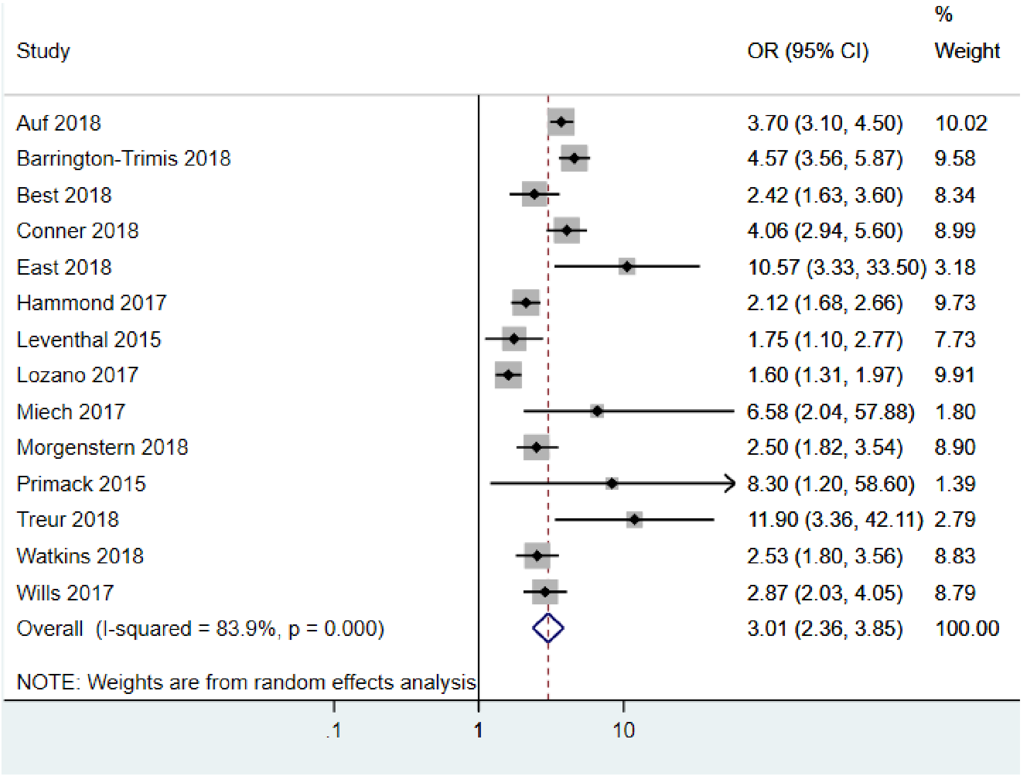

Figure S6. Forest plot for the adjusted association between e-cigarette use and later smoking among studies including under 18-year olds.

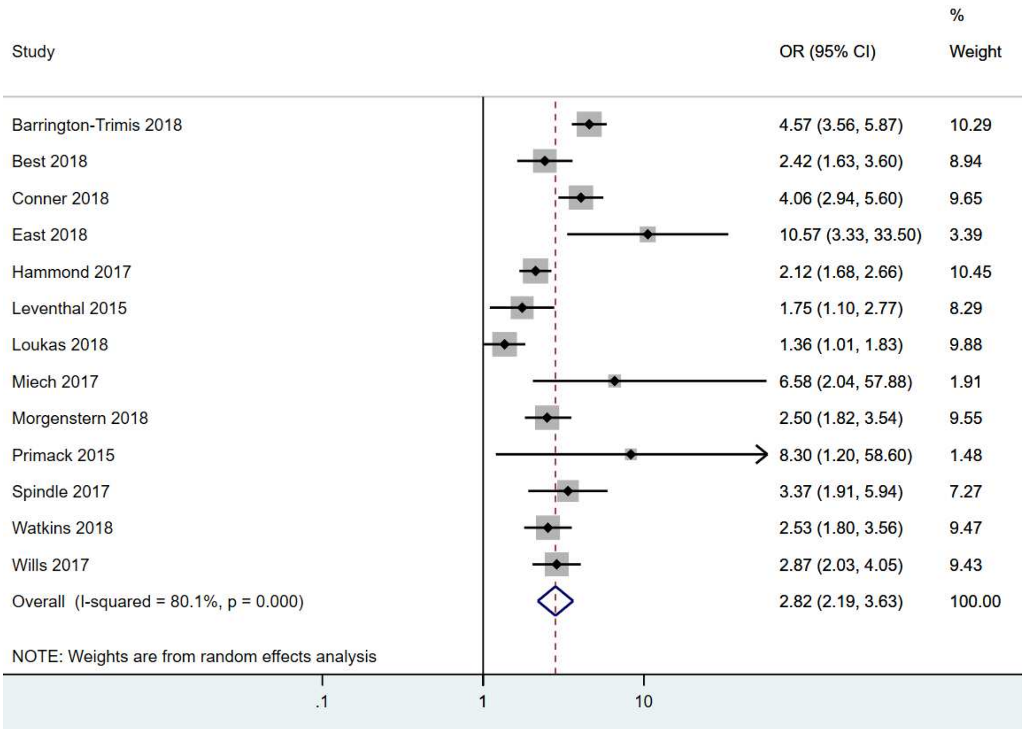

Figure S7. Forest plot for the adjusted association between e-cigarette use and later smoking among studies of good quality.

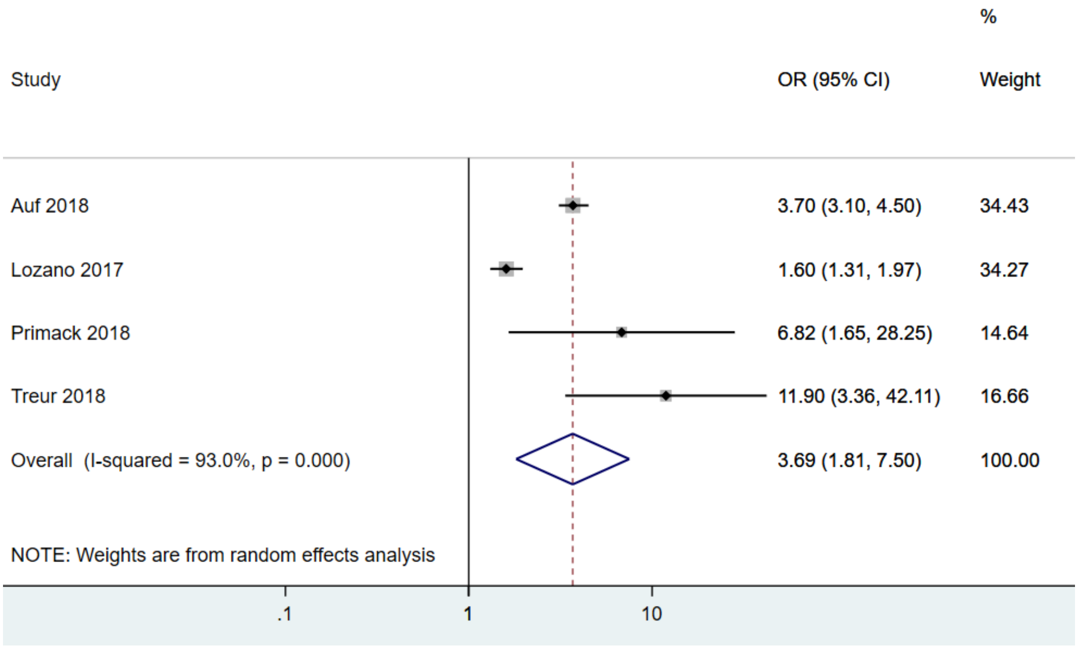

Figure S8. Forest plot for the adjusted association between e-cigarette use and later smoking among studies of fair/poor quality.

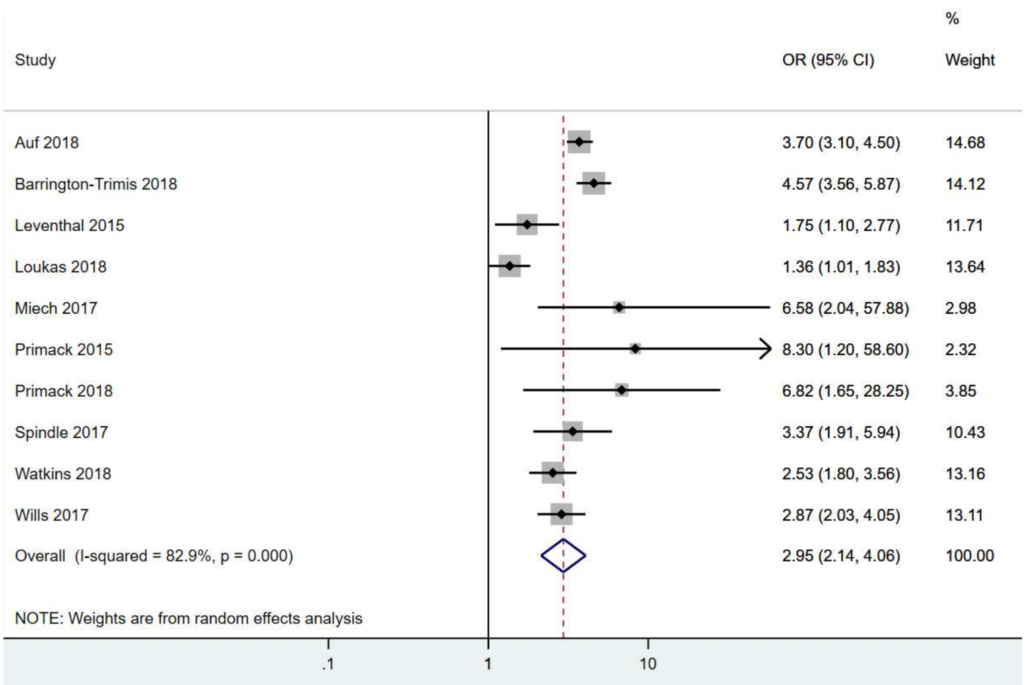

Figure S9. Forest plot for the adjusted association between e-cigarette use and later smoking among US studies.

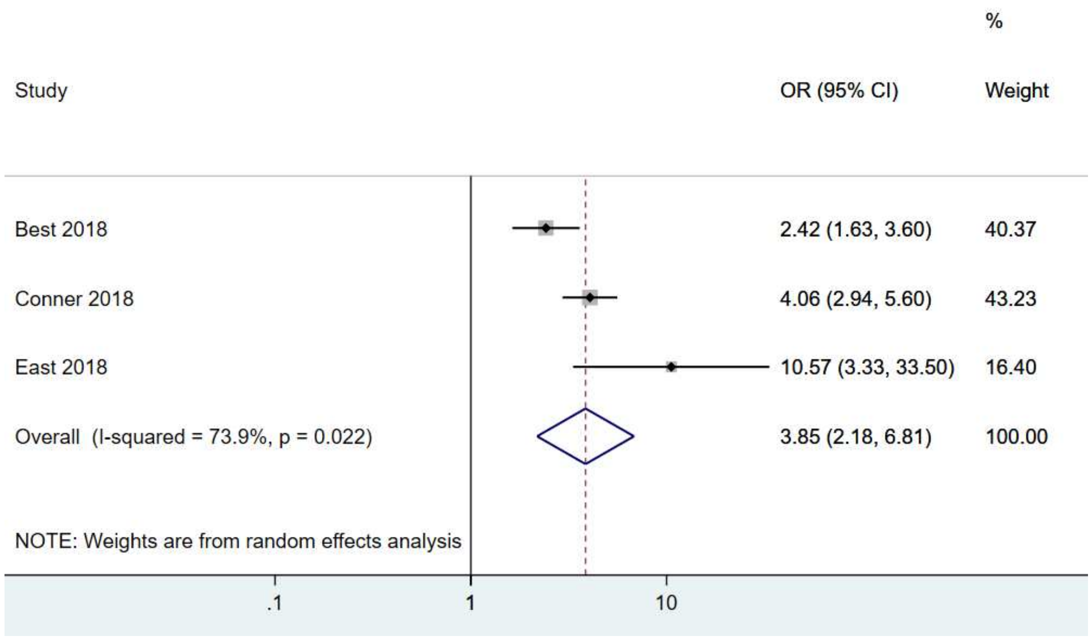

Figure S10. Forest plot for the adjusted association between e-cigarette use and later smoking among UK studies.

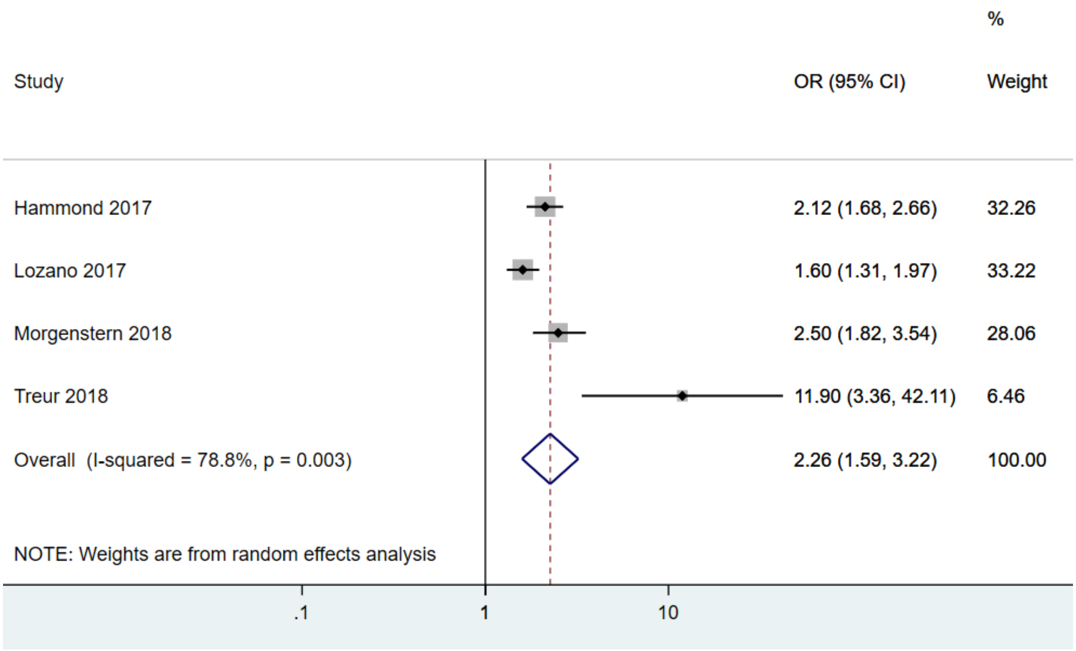

Figure S11. Forest plot for the adjusted association between e-cigarette use and later smoking among studies outside the UK and US.

Supplementary Tables

Table S1. Characteristics of included studies.

Study	Study size	Mean age* (range)**	Sex (% male)	Exposure	Outcome	Study type	Location of study	Follow up period (months)	Covariates included
Auf et al. (2018)	39,718	14.5 (12-19)	N/A	Ever-vapers	Ever smoking	Cross-sectional	USA	24	Age, race/ethnicity, gender, peer influence, household e-cigarette use and household use of outcome product
Barrington-Trimis et al. (2018)	6,258	N/A (grade 9-12)	N/A	Ever-vapers	Ever, frequent, and infrequent smoking	Longitudinal	USA	6-18	Gender, race/ethnicity, baseline grade in high school and study (random effect for school)
Best et al. (2018)	2,125	14.4 (11-12)	N/A	Ever-vapers	Ever smoking	Longitudinal	UK	12	Sex, age, ethnicity, and school
Conner et al. (2018)	1,726	13.18 (13-14)	48%	Ever-vapers	Ever smoking	Longitudinal	UK	12	Sex, family smoking, friends' smoking, intentions, attitudes, norms, perceived behavioural control, self-efficacy, and free school meals
East et al. (2018)	1,152	N/A (11-18)	46%	Ever-vapers	Ever smoking	Longitudinal	UK	4-6	Age, gender, school performance, problem behaviour, monthly alcohol use, smoking susceptibility, e-cigarette susceptibility, some friends smoke, some friends use e-cigarettes, at least one parent smokes, at least one parent uses e-cigarettes, sibling(s) smoke, sibling(s) use e-cigarettes and perceived public approval of e-cigarettes.

Hammond et al. (2017)	17,318	N/A (grade 9-12)	47%	Current vapers	Ever smoking	Longitudinal	Canada	12	Age, sex, race/ethnicity, and spending money
Leventhal et al. (2015)	2,530	14.1 (grade 9)	47%	Ever-vapers	Recent smoking	Longitudinal	USA	18	Age, sex, race/ethnicity, parental education, family living situation, family history of smoking, peer smoking, depressive symptoms, impulsivity, use of non–nicotine or tobacco substances, delinquent behaviour, susceptibility to smoking, and smoking outcome expectancies
Loukas et al. (2018)	2,558	19.71 (18-25)	33%	Ever-vapers	Ever smoking	Longitudinal	USA	20	Sex, age, race, type of college attended, susceptibility to smoking, family-of-origin tobacco use, friend cigarette use, and other tobacco use
Lozano et al. (2017)	4,695	N/A (11-13+)	48%	Ever-vapers	Ever smoking	Longitudinal	Mexico	18	Age, sex, parental education, parent smoker, sibling smoker, smoking among close friends, sensation seeking, trial of alcohol, trial of drugs, and internet tobacco product advertising
Miech et al. (2017)	347	Grade 12 (N/A)	44%	Current vapers	Ever smoking	Longitudinal	USA	12	Sex, race, parental education, baseline levels of marijuana use and binge drinking.
Morgenstern et al. (2018)	4,163	15.61 (14-18)	N/A	Ever-vapers	Ever smoking	Longitudinal	Germany	6	Sex, age, federal state, school type, migration background, school leaving qualification of parents, SES, sensation seeking, impulsivity, anxiety sensitivity, hopelessness, extraversion, agreeableness, conscientiousness, neuroticism, openness, alcohol ever, binge drinking ever, cannabis ever, other illegal drugs ever and

									participation in the “Keep a Clear Head” program.
Primack et al. (2015)	728	N/A (16-26)	46%	Ever-vapers	Ever smoking	Longitudinal	USA	12	Sex, age, race/ethnicity, maternal education level, sensation seeking, parental smoking, and smoking among close friends
Primack et al. (2018)	1,506	N/A (18-30)	39%	Ever-vapers	Ever smoking	Longitudinal	USA	12	Age, sex, race, ethnicity, education level, self-esteem, sensation seeking, rebelliousness, yearly household income, living situation and relationship status
Spindle et al. (2017)	2,316	18.5 (N/A)	38%	Ever and current vapers	Current smoking	Longitudinal	USA	12	Sex, age, race/ethnicity, depression, anxiety, negative urgency, positive urgency, lack of premeditation, lack of perseverance, sensation seeking, stressful life events, peer deviance, and other tobacco use
Treur et al. (2018)	6,819	13.8 (11-17)	52%	Ever-vapers	Ever smoking	Longitudinal	Netherlands	6	Age, sex, educational attainment and composite score of smoking propensity
Watkins et al. (2018)	10,348	14.3 (12-17)	51%	Ever and current vapers	Current smoking	Longitudinal	USA	12	Sex, age, race/ethnicity, parental educational, urban residence, sensation seeking, alcohol use, living with tobacco user, frequency of noticing of tobacco warnings, receptivity to tobacco advertising, and season
Wills et al. (2017)	1,141	14.7 (14-16)	47%	Ever-vapers	Ever smoking	Longitudinal	USA	12	Age, sex, race/ethnicity, family structure, parental education, parental support, parental monitoring, sensation seeking, rebelliousness, and clustering within school

*Age reported in years except where grade is stated. Grade reported where it was provided in the study, but actual age was not stated. **At baseline.

Table S2. Within study risk of bias and relevant Bradford-Hill criteria for causality

Study	Newcastle -Ottowa Scale Quality Rating	Strength of adjusted odds ratio (OR)	Adjusted for more than basic demographics	Longitudinally assessed	Frequency/ length of use/nicotine content taken into account	Number of BH criteria met out of 4
Auf et al. (2018)	Poor	Strong	Yes	No	No	2
Barrington-Trimis et al. (2018)	Good	Strong	Yes	Yes	No	3
Best et al. (2018)	Good	Strong	Yes	Yes	No	3
Conner et al. (2018)	Good	Strong	Yes	Yes	No	3
East et al. (2018)	Good	Strong	Yes	Yes	No	3
Hammond et al. (2017)	Good	Strong	No	Yes	No	2
Leventhal et al. (2015)	Good	Weak	Yes	Yes	No	2
Loukas et al. (2018)	Good	Weak	Yes	Yes	No	2
Lozano et al. (2017)	Fair	Strong	Yes	Yes	No	3
Miech et al. (2017)	Good	Strong	Yes	Yes	No	3
Morgenstern et al. (2018)	Good	Strong	Yes	Yes	No	3
Primack et al. (2015)	Good	Strong	Yes	Yes	No	3
Primack et al. (2018)	Poor	Strong	Yes	Yes	No	3
Spindle et al. (2017)	Good	Strong	Yes	Yes	No	3
Treur et al. (2018)	Poor	Strong	Yes	Yes	Yes	4
Watkins et al. (2018)	Good	Strong	Yes	Yes	No	3
Wills et al. (2017)	Good	Strong	Yes	Yes	Yes	4

Note: Thresholds were applied to convert the Newcastle-Ottawa scales to Agency for Health Research and Quality standards (whereby a good quality rating indicates low risk of bias and a poor rating indicates high risk of bias). Good quality = 3 or 4 stars in selection domain AND

1 or 2 stars in comparability domain AND 2 or 3 stars in outcome/exposure domain. Fair quality = 2 stars in selection domain AND 1 or 2 stars in comparability domain AND 2 or 3 stars in outcome/exposure domain. Poor quality = 0 or 1 star in selection domain OR 0 stars in comparability domain OR 0 or 1 stars in outcome/exposure domain.
*Odds ratios described as strong if more the 2 and weak if less than or equal to 2.

Khouja JN, *et al. Tob Control* 2020; 30:8–15. doi: 10.1136/tobaccocontrol-2019-055433
